# Traditional Knowledge to Contemporary Medication in the Treatment of Infectious Disease Dengue: A Review

**DOI:** 10.3389/fphar.2022.750494

**Published:** 2022-03-14

**Authors:** Mamta Dhiman, Lakshika Sharma, Abhishek Dadhich, Poonam Dhawan, M. M. Sharma

**Affiliations:** ^1^ Department of Biosciences, Manipal University Jaipur, Jaipur, India; ^2^ School of Science, Nirwan University, Jaipur, India

**Keywords:** dengue, traditional medicine, plant medicaments, bioactive compound, immunomodulatory response

## Abstract

Dengue has become a worldwide affliction despite incessant efforts to search for a cure for this long-lived disease. Optimistic consequences for dengue vaccine are implausible as the efficiency is tied to previous dengue virus (DENV) exposure and a very high cost is required for large-scale production of vaccine. Medicinal plants are idyllic substitutes to fight DENV infection since they constitute important components of traditional medicine and show antiviral properties, although the mechanism behind the action of bioactive compounds to obstruct viral replication is less explored and yet to be discovered. This review includes the existing traditional knowledge on how DENV infects and multiplies in the host cells, conscripting different medicinal plants that obtained bioactive compounds with anti-dengue properties, and the probable mechanism on how bioactive compounds modulate the host immune system during DENV infection. Moreover, different plant species having such bioactive compounds reported for anti-DENV efficiency should be validated scientifically *via* different *in vitro* and *in vivo* studies.

## 1 Introduction

Mosquito-borne diseases cause illness in approximately 700 million people per year globally (Genoud et al., 2018). Dengue is one of the mosquito-borne emerging viral disease endemics prominent in many urban areas of tropical countries. A considerable rise in the dengue infection incidences is noticed all over the world in recent decades. Due to its asymptomatic to mild infectious nature, actual numbers of dengue cases remain unregistered or wrongly diagnosed as other fever infections (Waggoner et al., 2016). According to the World Health Organization report, about 390 million cases of dengue virus (DENV) infections occur annually, of which nearly 96 million find medical treatment and about 20,000 individuals (mostly children and aged individuals) fail to survive the dengue infection (Bhatt et al., 2013; Saxena et al., 2017; World Health Organization 2021a). Remarkably, among 129 countries having a risk of dengue infection, Asia has almost 70% of the total infection load (Brady et al., 2012; Bhatt et al., 2013). Dengue, an arthropod-carried viral infection, is transmitted into humans by female *Aedes* mosquitoes (*Ae. aegypti* or, to a lesser extent, by *Ae. albopictus* and *Ae. polynesiensis*) (Cao-Lormeau 2009). Common symptoms in dengue infection are mild dengue fever to severe dengue hemorrhagic fever (i.e., DF and DHF), rashes, headache, vomiting, severe headache, low white blood cell count, joint and muscle pain, swollen glands, nausea, pain behind the eyes, and fever with dengue shock syndrome (DSS) (Murphy and Whitehead 2011; World Health Organization 2021a).

The natural reserve has offered various resources to mankind to combat various infectious diseases. Plants are the most frequently utilized natural sources among the local biodiversity and are linked to folk’s day-to-day needs (Robinson and Zhang 2011). Additionally, plant-based medicaments are in considerable demand because of their safer use and non-toxic nature, and because they are less harmful in comparison to synthetic treatments (Abd Kadir et al., 2013). Multiple uses, such as consumption as food, ethnomedicinal applications, cultural aspects, and sacred faith, are associated with their utilization (Svanberg and Berggren 2019; Fongnzossie et al., 2020). Traditional knowledge of plants that was commonly practiced among tribal or ethnic populations for their healthcare has now gained considerable attention from the modern populace. Nowadays, people in developing (60%–90%) as well as developed countries (23%–80%) are using ethno-medicines as the primary healthcare regimen (Borah and Prasad 2017; Sharma and Pareek 2021). Plant biodiversity used as medicaments is of utmost importance throughout human history, which has led to the accumulation of significant information in the form of scientific research and its validation (Pasa 2011). These conventional plant-based healthcare remedies led the Ayurvedic, Unani, Chinese, and Egyptian medical systems to classify various medicinal herbs (based on color, aroma, shape, flavor, and astronomic and magical attributes) (Larocca et al., 2021). Traditional use and ethnobotanical knowledge about many plant species in treating various ailments along their chemical validation through scientific research can be promising aspects in the development of contemporary medication to treat arboviral infections like dengue. Also, a plant-based antiviral product assures a more possible choice in combating dengue infection, which may be replacement of inadequate drugs with side effects (Bahuguna et al., 2019). WHO in the 1970s has effectively executed the spread of traditional knowledge *via* implementing Traditional Medicine Program, and consequently, following such initiative (taking into consideration the traditional knowledge and scientific research), the associated nations have been redeveloping and improving their public health systems (Larocca et al., 2021). Development of an efficient and safer anti-dengue vaccine is a challenging task, and because of its secondary infection (second time DENV serotype infection), it is linked to the serious clinical manifestations (Toman 2018; Redoni et al., 2020). Based on recent research, various plants and their derivatives have shown anti-dengue properties; among those, flavonoids are the most popular candidates, having the ability to supplement encouraging scope in the existing struggle of drug discovery (Carneiro et al., 2016). Some of the important flavonoids recently reported are discussed with various activities, such as antioxidative, anti-inflammatory, antitumoral, antiviral, and antibacterial effects (Raekiansyah et al., 2018; Deng et al., 2020b; Daneshzadeh et al., 2020; Macedo et al., 2020; Wang et al., 2020). In the present review, a brief idea of the role of these phytoconstituents has been described against dengue infections (Jayadevappa et al., 2020). In addition, the mechanism of action with future possibilities as contemporary medicaments of plant metabolites that endorses traditional knowledge is also included.

## 2 Epidemiology of Dengue

Dengue is a mosquito-borne viral infection, found in tropical and sub-tropical climates worldwide, mostly in urban and semi-urban areas, transmitted by *Aedes aegypti* and *Aedes albopictus*, known as the primary and secondary dengue vectors. A range of diseases has been transmitted via *Ae. aegypti* including yellow fever, chikungunya, zika, and, most importantly, DENV (Silva et al., 2020). DENV was first reported in the 1950s, during dengue epidemics in Thailand and Philippines. Since then, the spread of dengue has extensively widened from South Asian countries to African and South American countries. Over several decades, different countries of continental America conducted a program that aimed to eradicate *Ae. aegypti*. The program was proposed and commenced *via* the Pan American Health Organization (PAHO) in 1946 (Pinheiro and Rodrigues 1999). Venezuela, a country on the northern coast of South America, reported dengue fever outbreaks during the 1960s, when almost all South American countries had eradicated *Ae. aegypti* (Pinheiro and Corber 1997). Furthermore, during the 1970s, DENV-2 and DENV-3 were the main causative serotypes for dengue fever epidemics in Colombia, a country that had attained eradication of the vector during the PAHO program (Pinheiro 1997). In the 1980s, enhanced rate of dengue cases escorted the spreading of *Ae. aegypti* vector, and during this period, another efficient vector, the Asian mosquito, *Ae. albopictus*, was introduced in the region (Gubler 1989). In the last 5 decades, the American continent has been massively affected by dengue and an approximately 30-fold rise in disease level was recorded, where almost 390 million cases of dengue with 96 million medical manifestations were reported annually (Bhatt et al., 2013). In 2013, America has declared approximately 2.3 million new severe dengue cases, which was an alarming situation in viral infections (Salles et al., 2018).

*Ae. albopictus* was mostly found in Asia and further expanded over different countries of European and North American regions, having highly adaptive properties to survive extreme environments and therefore able to survive in cooler regions as well. The probability of an upsurge of arthropod-borne viruses is based on the abundance of vectors involved in transmission as well as the susceptibility of immune-deficient people. Hence, there is a necessity to develop an efficient way to control its population and the spread of the disease. Besides, several other causes such as increased urbanization, ecological changes, migration of people, exchange of goods, and biological contests (e.g., development of resistance for virus) stimulate the spread of disease to new regions (Chaudhry 2019).

## 3 Replicative Cycle and Pathogenesis of Virus

**FIGURE 1:**
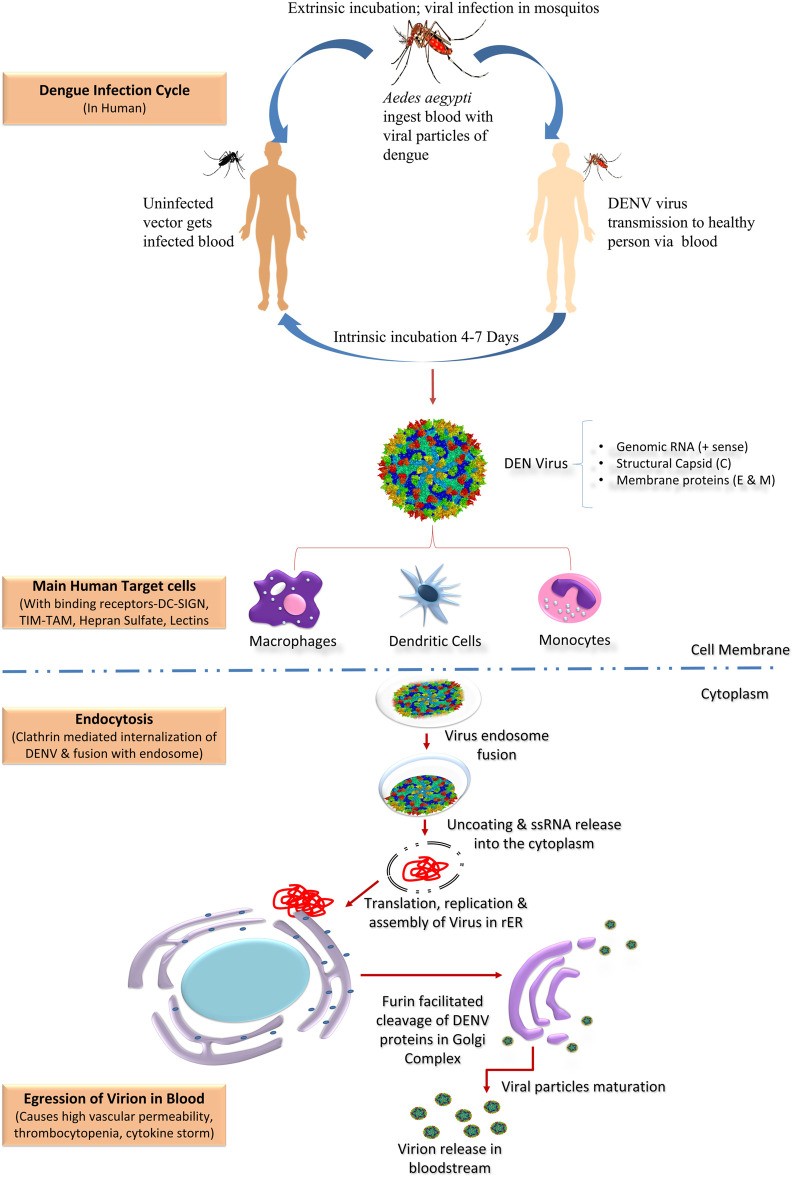
Dengue infection cycle in human body.

The genomic structure of DENV, approximately 10.7 kb in size, consists of ssRNA with +ve polarity (Chambers et al., 1990). Besides, the translation of viral genome was completed by three different structural proteins, i.e., Envelop (E), Capsid (C), and Pre-membrane (PrM), and seven non-structural (NS) proteins, NS1, NS2a, NS2b, NS3, NS4a, NS4b, and NS5. These proteins contribute to viral replication (Bartenschlager and Miller 2008; Xie et al., 2017). The infective form of dengue virion possesses a core of glycoprotein and ssRNA genome enclosed by icosahedral nucleocapsid (Akhtar 2019). Firstly, infected mosquito feed on host and DENV gets transmitted *via* injecting its saliva into the host. In the meantime, when half-length proboscis is inside the dermis, the vector releases DENV plaque-forming units (nearly 50,000) and causes infection in contiguous skin cells, i.e., Langerhans and keratinocytes (Marcial-Juárez et al., 2017; Schneider et al., 2021). Dendritic cells enter lymphatics after capturing DENV virions or antigens and transport them to local draining lymph nodes. DENV is also suggested to reach draining lymph nodes *via* the lymphatic flow in a cell-free manner (Yam-Puc et al., 2016). The E protein of DENV assists the binding between DENV and the cell membrane receptor. Receptor-mediated endocytosis is the principal way *via* which the virus is able to enter the host; a resultant sac-like structure is formed known as an endosome, depending on pH. Virus acutely penetrates and fuse with the membrane of the endosome due to the various irretrievable morphological reorganization as the endocytic vesicles become more acidic, followed by nucleocapsid opening and genetic material (RNA) release into the cytoplasm. RNA succeeds in translating into ribosomes allied to the ER (endoplasmic reticulum) by using the infected cell’s machinery; subsequently, the viral polyprotein is cleaved by cellular and viral proteases (Uno and Ross 2018). The newly synthesized RNA is wrapped in the capsid proteins (nucleocapsid), enters the host rER, and ultimately encloses the ER membrane. Further structure proteins (M and E) surround the ER-enveloped nucleocapsid forming immature virus. Subsequent processing in the Golgi apparatus results in the formation of the infectious form of the virus (Aruna 2014). Afterwards, it reaches the lymph node and further disseminates to other body organs. The virus incubation period may vary from 3 to 14 days based on the literature available (Chan and Johansson 2012). A general overview of the DENV infection cycle in humans is shown in Figure 1. After completion of the virus incubation period, the mosquito is capable of spreading the virus in the course of its life. *Ae. aegypti* feeds mostly on human blood and is a daytime feeder, efficiently causing infection in the early morning, and by evening, it infects multiple people during each suckling period (Weissenböck et al., 2010). The infecting efficiency of DENV for different cells including liver, blood vessels (endothelium), immune system, and retinal cells has been reported in the past (Carr et al., 2017; Begum et al., 2019).

### 3.1 Dengue Infection

The occurrence of dengue has intensely spread worldwide in recent decades with a majority of asymptomatic cases (World Health Organization, 2021a). The infection efficiency can vary from subclinical disease to acute flu-like symptoms. Three different categories of symptomatic dengue, namely, dengue fever (DF), dengue hemorrhagic fever (DHF), and undifferentiated fever, were classified by WHO in 1997. Due to the changing epidemiology of infection, it was difficult to fulfill the suggested WHO guidelines (for dengue), and thus, classification was re-evaluated (World Health Organization 2009). In the new classification system, DHF was further categorized up to four levels based on severity of infection, with grades III and IV as DSS. Dengue asymptomatic cases were divided into two different categories: with warning and without warning signs of severe dengue (World Health Organization 2009). Dengue is associated with several complications, which are characterized by headache, appetite loss, high fever, retro-orbital pain, vomiting, abdominal pain, diarrhea, minor bleeding, rash, fatigue, etc. Severe dengue is classified by the presence of severe plasma bleeding, organ impairment, or dengue shock conditions (Khan and Bhutta 2016). It is also designated as “break-bone fever” (Rigau-Pérez 2006; Esler 2009). The severity of infection can be strongly provoked by numerous factors, such as virus serotypes, immunity power, and genomic background of host, among others (Rico-Hesse 2010; Dussart et al., 2012). Moreover, four different serotypes of viruses (DENV-1, DENV-2, DENV-3, and DENV-4) of the Flaviviridae family were deliberated as causal forms of dengue (Guzman and Istúriz 2010). Although every serotype has an equal potential to cause infection among the four serotypes of dengue, serotype variances that only affect pathogenesis such as DENV-2 have been associated with severe infection (Halstead 1988; Vaughn et al., 2000).

Recovery from infection provides enduring immunity against that serotype, although immunity for other serotypes known as cross-immunity is temporary and partial. Consequent contagions (secondary infection) by different serotypes increase the threat of emerging severe dengue. Dengue has discrete epidemiological forms, allied with the four serotypes of the virus (Halstead 2002; Rico-Hesse 2010; Dussart et al., 2012; Kim and Hwang 2020). Different studies based on the data analysis of infection status among the Indian population have been reported (Table 1). Despite this, numerous cases have been found in different regions of India, which is still unexplored in terms of proper data illustrations.

**TABLE 1:** Dengue seroprevalence by age, reported in some studies from India.

Padbidri et al. (2002) (*n* = 717)		(Garg et al., 2011) (*n* = 2,558)		(Rodríguez-Barraquer et al., 2015) (*n* = 800)		(Murhekar et al., 2019) (*n* = 12,300)		(Shah et al., 2017) (*n* = 700)	
Age (years)	Seroprevalence (%)	Age (years)	Seroprevalence (%)	Age (years)	Seroprevalence (%)	Age (years)	Seroprevalence (%)	Age (years)	Seroprevalence (%)
0–9	47.60%	5	40.70%	9-May	77%	8-May	33.00%	0–10	13.70%
19-Oct	24.00%	6	50.90%	14-Oct	90.30%	17-Sep	34.70%	20-Nov	30.10%
20–29	26.80%	7	58.60%	15–19	91.70%	18–45	32.30%	21–30	45.10%
30–39	25.00%	8	67.40%	20–29	96.30%	—	—	31–40	59.70%
≥40	23.30%	9	70.80%	30–40	98.80%	—	—	≥40	64.10%
—	—	10	73.40%	—	—	—	—	—	—
Total	25.4 (95% CI = 22.3–28.7)	—	59.6 (95% CI = 57.7–61.5)	—	93 (95% CI = 91.1–94.6)	—	-—	—	42.8 (CI = 40.9–44.7)

## 4 Mode of Transmission

*Aedes* spp. mosquitoes (*Ae. aegypti* and *Ae. albopictus*) are the main source of dengue transmission. Recently, infection dispersed in almost all parts of tropical and subtropical regions and adapted to urban environments, mainly *Ae. aegypti* (Lambrechts et al., 2010). Among two different species, *Ae. aegypti* has been reported to have superior efficiency as a transmission vector; meanwhile, in urban environments, DENV is capable to endure its life cycle between humans and transmitter organisms (Lambrechts et al., 2010; Weissenböck et al., 2010). Human blood is the primary feeding source of *Ae. aegypti*, while *Ae. albopictus* (sylvatic strain) feeds on avian species as well as a variety of mammals (European Centre for Disease Prevention and Control 2018). Sylvatic strains are not that risky compared to “standard” dengue infection (Diallo et al., 2003; Vasilakis et al., 2008). In the initial stage of transmission, uninfected mosquitoes attack (susceptible to dengue) while feeding on host blood. In this process, viral particles are transferred into the mosquito midgut and start replicating and thus infecting the body cavity (hemocoel). From the hemocoel, the virus eventually makes its way to the salivary glands. Next, after completing the incubation period (approximately 2 weeks), the infected mosquito can disseminate the virus *via* salivary glands (Coudeville et al., 2009; Guzman et al., 2016).

## 5 Impact of Dengue Infection Globally and in India

**FIGURE 2:**
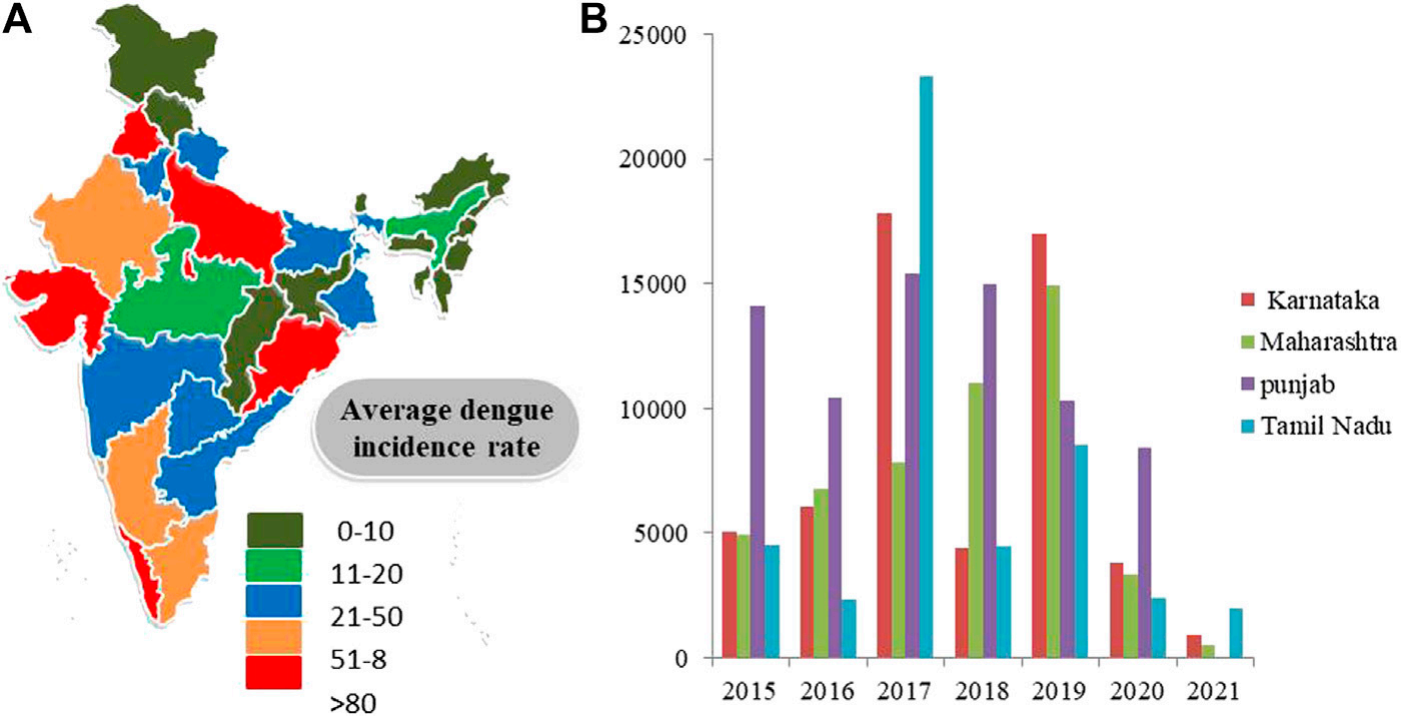
**(A)** Dengue cases in different regions of India 2021. **(B)** State-wise distribution of dengue cases (*y*-axis) in four states of India from 2015 to 2021 (*x*-axis) (Source: National Vector Borne disease Control Programme).

**FIGURE 3:**
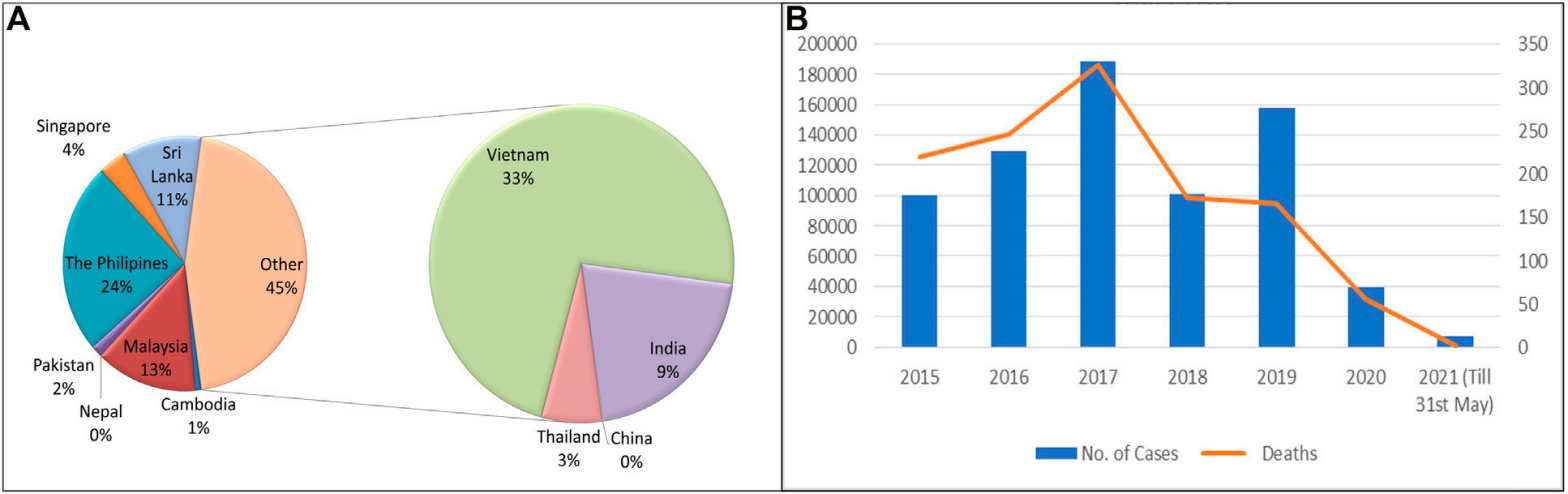
**(A)** Comparative analysis of dengue incidences in different Asian countries (Source: WHO and European Center for disease Prevention and Control). **(B)** Number of dengue cases and deaths in India (2015–2021 May). (Source: National Vector Borne disease Control Programme).

Dengue has emerged as one of the most important mosquito-borne, flaviviruses diseases, deceptively intensifying as a global health issue (Zeng et al., 2021). Dengue has been designated as the topmost worldwide health risk by WHO (Kumar et al., 2017; Biswal et al., 2019). A comparison of prior approximations of entire global dengue contagions has been illustrated. Average incidences in India with maximum infected states (Karnataka, Punjab, Tamil Nadu, and Maharashtra) are shown (Alagarasu et al., 2021) (Figure 2). Moreover, dengue infection frequency of different Asian countries and their percentage contribution have been determined where India was found to comprise 9% of total dengue incidences, higher than Pakistan, Singapore, China, Thailand, Nepal, and Cambodia, while Vietnam and Philippines are the leading countries and recorded the highest number of dengue cases in 2021 (till May) (Figure 3A) (European Centre for Disease Prevention and Control 2018; World Health Organization 2021b). Also, dengue infection status recorded in the past 7 years in India can be seen where the average infection was recorded to be the maximum in 2017 (Figure 3B) (NVBDCP, 2021).

## 6 Plant Medicaments Used Based on Traditional Knowledge

Plants have been the main remedial source to cure a variety of ailments since ancient times. Ancestral folks empirically isolate medicinal plants for medical home remedies and pass this knowledge from generation to generation till the present day. Ethnobotany has confirmed the necessity for conserving such information in societies that utilize plants in the treatment of different types of diseases (Gu et al., 2014; Mussin and Giusiano 2020). However, it is critical to note that these practices of exploiting plant medicaments, without scientific understanding, have led to placing various plants in the IUCN Red List consisting of threatened species (Trujillo-Correa et al., 2019). The dengue treatment period is suggested to be 10–15 days, which may vary according to the infection level of severity, the regimen carried out during treatment, the conditions, and the response of the organism (Yogarajalakshmi et al., 2020). Different plant-based formulations are reported to be used in various affected parts of India against dengue and have also been approved through scientific research, although limited studies in this reference have been recorded in India (Deep et al., 2018). There are many plant species such as *Curcuma longa* L., *Lonicera japonica* Thunb.*, Acorus calamus* L., *Carica papaya* L., *Euphorbia hirta* L., *Tinospora sinensis* (Lour.) Merr., and *Sambucus canadensis* L. used globally as ethnomedicine against dengue fever (Ichsyani et al., 2017; Lee et al., 2017; Deep et al., 2018; Yao et al., 2018; Larocca et al., 2021) (Table 2). In another report, plants from a diverse group of plant families (31 in total with 54 species) are reported to exhibit anti-dengue activity based on traditional knowledge, where major representatives reported were Lamiaceae (10.5%), Asteraceae (9.9%), Aristolochiaceae, and Loganiaceae (each 7.2%) families (Saleh and Kamisah 2021). Plants revealed by the local population to exhibit anti-dengue activity included *Baccharis trimera* (Less.) DC., *Aristolochia surinamensis Willd.*, *Momordica charantia* L., *Dysphania ambrosioides* (L.) Mosyakin and Clemants, *Ocimum gratissimum* L., *Strychnos pseudoquina* A. St.-Hil., and *Stachytarpheta cayennensis* (Rich.) Vahl (Pongthanapisith et al., 2013; Sharma et al., 2020; Larocca et al., 2021). Although many of these plant species do not have any scientific evidence as suggested in the available literature, their ethnomedicinal potential may lead to the real success in treating arboviral diseases like dengue (Pilla et al., 2006; Mendoza et al., 2020). However, limited literature presenting scientific validation of these plants showing anti-dengue activity is available. Traditional use of *C. papaya* leaf juice has revealed very promising outcomes in treating dengue disease (Prakash Kala, 2012; Sarala and Paknikar 2014), which is further authorized through scientific evidence demonstrating lower cytotoxic and efficient anti-DENV effects (Saptawati et al., 2017; Sharma et al., 2019; Sarker et al., 2021). Similarly, the use of *Psidium guajava* L. as folkloric medication has also been cited in literature, which enhances thrombocyte count in dengue infection (Berlian et al., 2017). Traditional medicinal values being the foundation for advanced scientific research lead to the revelations of possible mechanisms behind the anti-dengue activities that suggest a promising role of phytoconstituent-associated antiviral activities *via* inhibition of viral cell adhesion, replication, etc. (Saptawati et al., 2017; Dewi et al., 2020). Other traditionally used plants such as *Ocimum tenuiflorum* L. (Tulsi), *Tinospora sinensis* (Lour.) Merr. (Giloy leaves and stems), *Trigonella gladiata* M. Bieb. (fenugreek leaves), *Azadirachta indica* A. Juss. (Neem leaves), *Cymbopogon citratus* (DC.) Stapf (Lemon grass), and *Psiloxylon mauritianum* (Bouton ex Hook. f) Baill. with potential ethnopharmacological uses in treating dengue still need clarification about their action mechanism technically (Parida et al., 2002; Abd Kadir et al., 2013; Ling et al., 2014; Singh et al., 2016; Deep et al., 2018; Arunabha 2019; Clain et al., 2019; Rosmalena et al., 2019; Paul et al., 2021). There are very few reports validating the antiviral activities of *A. indica*. According to these reports, aqueous *A indica* leaf extracts and Gedunin and Pongamol (neem constituents) are capable of repressing DENV-2 serotype by targeting viral replication (Parida et al., 2002) and targeting human as well as viral proteins (responsible for viral attachment) (Rao and Yeturu 2020). *Quercus lusitanica* Lam. and *Gastrodia elata* Blume have also been approved to have an antiviral response against DENV-2 replication and DENV multiplication cycle, respectively (Rahman et al., 2006; McDowell et al., 2010; Ahmad et al., 2011). Also, the polyphenol-rich extract of *Aphloia theiformis* (Vahl) Benn. has recently been reported against ZIKV and DENV infections, which prohibits viral entry (Clain et al., 2018).

**TABLE 2:** List of plants studied for anti-dengue response based on ethnopharmacological use.

Sr. No	Plant/Common name/Family		Geographical location	Part Used	Studied Model, DENV Serotype	Optimum dosage	Mode of action	Used cell line/animal/human model	Citation				
		Section1 *: In vitro studies*											
1	*Acacia catechu*. (L.f.) Willd./Khair/Fabaceae		Maharashtra, Madhya Pradesh, Gujarat, Tamil Nadu, Uttar Pradesh, Karnataka, Andhra Pradesh, and Rajasthan	Dried powder of fruits	DENV 1–4	IC_50_ values 1.54 μg/ml and 0.18 μg/ml	Extract contains peptides, subdued DENV infection in the initial phase of infection *via* decrease foci formation and level of intracellular envelope proteins were also decreased in all serotypes of DENV.	Vero cells (kidney epithelial cells isolated from African green monkeys) and Huh7 cells (hepatocellular carcinoma cell line)	Panya et al. (2019); Panya et al. (2020)				
							Most effective peptide (designated Pep-RTYM) interacted with DENV particles and inhibited cellular entry						
2	*Acorus calamus* L./sweet flag, sway or calamus/Acoraceae		Europe, China, northern Asia Minor, Indonesia, southern Siberia, in southern Russia, Japan, Sri Lanka, Australia, Burma, as well as southern Canada and Northern United States	Roots	DENV2	Tatanan A, EC_50_ = 3.9 μM	Potentially affect DENV2, treatment constrained the initial steps of RNA synthesis as well as post translation modifications	Mosquito larva C6/36 cells were for DENV2 replication and Mouse kidney fibroblast cells (BHK-21)	Yao et al. (2018)				
3	*Andrographis paniculata* (Burm.f.) Nees/Green chiretta/Acanthaceae		Widely cultivated in Southern and South eastern Asia, Malaysia	Methanolic extract	DENV 2,4	Andrographolide, the maximum non-toxic dose 15.62 μg/ml	—	C6/36 cell line for DENV replication	Kaushik et al. (2021)				
4	*Basilicum polystachyon* L. Monecch/Musk Basil/Lamiaceae		Asia, Asterids, Africa, Borneo, Indochina Australia, India, China, Indian Ocean	Whole plant	DENV serotype not mentioned	IC_50_ = 1.4 ± 2/1 μM	—	Vero cells (African green monkey kidney)	Tan et al. (2019)				
5	*Cissampelos pareira* L./Velvet leaf/Menispermaceae		Western and Eastern Cape Provinces Sandy slopes and scrub of the Northern, northwards into Namibia	Aerial parts	DENV-1–4	IC_50_ values 100, 125, 78, and 100 μg/ml, respectively	It obsessed the efficiency to downregulate the synthesis of TNF-α, a type of cytokine allied with acute dengue disease	Mosquito cell line C6/36 and monkey kidney cell lines LLCMK2	Sood et al. (2015)				
6	*Curcuma longa* L./Turmeric/Zingiberaceae		Originate from South or Southeast Asia, most probably from Vietnam, western India, or China	Not mentioned	DENV-2	IC_50_ = 17,91 μg/ml CC_50_ = 85,4 μg/ml	-	Huh7it-1 cells	Ichsyani et al. (2017)				
7	*Cymbopogon citratus* (DC.) Stapf/Lemongrass/Gramineae		Indigenous to Sri Lanka and South India, recently cultivated in the tropical areas of Asia and America	Not mentioned	DENV-2	CC_50_ = 183.74 and EC_50_ = 29.37 μg/ml	After treatment viral replication inhibition increased	Antiviral activity in Huh7it-1 cell lines infected by DENV	Rosmalena et al. (2019)				
8	*Doratoxylon apetalum* (Poir.) Radlk./Sapindaceae		Native to Mascarene Islands	Aerial parts	DENV1–4	IC_50_ = 96.35, 16.75, 25.90, and 23.30 μg/ml for DENV1–4 individually	Extract-mediated DENV inhibition is allied to an erosion of infectivity	Human lung epithelial A549 cells, Vero cells, and human-derived Huh-7 hepatoma cells	Haddad et al. (2019)				
9	*Ficus septica* Burm.f./Hauli tree/Moraceae		Japan, Indonesia, Malaysia, Philippines, Solomon Islands, Papua New Guinea, and Taiwan	Stem fruit, heartwood, and leaves	DENV-1 and DENV-2	IC_50_ = 3.05–>100 μg/ml	—	DENV-1 766733A and DENV-2 PL046 (GenBank accession no. AJ968413.1)	Huang et al. (2017)				
10	*Oldenlandia uniflora* L./Geta Kola/Rubiaceae		Mostly found in Sri Lanka	Leaves, stems, and roots	DENV-2 NS2B-NS3pro	IC_50_ ≤ 100 μg/ml	—	Dengue NS2B-NS3pro	Rothan et al. (2014)				
11	*Kaempferia parviflora* Wall. ex Baker/krachai Dam/Zingiberaceae		—	Leaves and stems	DENV-2	—	-—	—	Umesh Kanna and Krishnakumar (2019)				
12	*Nephelium lappaceum* L./Rambutan/Sapindaceae		Southeast Asian native to the Malaysian–Indonesian region	Rind	DENV-2	IC_50_ = 1.75 μM	Restricts early phases of cell and virus interaction *via* inhibiting the attachment of virus with the binding of E-DIII protein	African Green Monkey kidney cells (Vero)	Abdul Ahmad et al. (2017)				
13	*Ocimum tenuiflorum* L./Tulsi, basil/Lamiaceae		Indigenous to Iran and India and currently cultivated in France, Egypt, Italy, Hungary, Morocco, and United States.	Whole aerial body	DENV-1	Maximum non-toxic dose: 23.44 μg/ml	*O. sanctum* unveiled antiviral efficacy for DENV-1 *via* inhibiting CPE formation and multiplication of virus	HepG2 cells	Ling et al. (2014)				
14	*Pavetta canescens* DC./Papari/Rubiaceae		E. Asia—India, Sri Lanka, and Philippines	Leaves	DENV-2	Least LC_50_ and LC_90_ values (5.968 and 7.493 μg/ml)	—	Monolayer culture of C6/C36 mosquito cell line	Pratheeba et al. (2019)				
15	*Psidium guajava* L./Common guava/Myrtaceae		Native to the Caribbean, Central America, and South America	Bark	DENV-2	Catechin, CC_50_ = 1,000.0; μg/ml EC_50_ = 7.8	—	Epithelial VERO cells and C6/36HT cells (from *Aedes albopictus* mosquito larvae)	Trujillo-Correa et al. (2019)				
16	*Schisandra chinensis* (Turcz.) Baill./Magnolia-vine, Chinese magnolia-vine/Schisandraceae		Indigenous to forests of Northern China and the Russian Far East and Korea	—	DENV-1, 2, 3, and 4	Schisandrin A, EC_50_ = 28.1 ± 0.42 μM	Isolated bioactive compound, restricted RNA replication and translation as well as ominously raised DENV-reduced IFN-α gene expression	DENV-infected Huh-7 cells	Yu et al. (2017)				
17	*Tarenna asiatica* (L.) Kuntze ex K. Schum./Bingi Papadi/Rubiaceae		Southern part of India, Sri Lanka, and Malaysia	Leaves	DENV serotype not mentioned	Tetracontane, LC_50_ and LC_90_ values (1.288 and 1.992 μg/ml)	—	Monolayer culture of C6/C36 mosquito cell line	Pratheeba et al. (2019)				
18	*Zostera marina* L./Eagrass or eelgrass/Zosteraceae		Northern Hemisphere as well as Australia, New Zealand, Southeast Asia, and southern Africa	Designed and provided by CernoFina, LLC (Portland, ME)	DENV-1–4	ZA, IC_50_ = 2.3 mM	—	Monkey kidney cell line LLCMK-2	Rees et al. (2008)				
		Section 2: *In vitro* gene or protein expression studies											
19	*Allium sativum* L./Garlic/Liliaceae		United States and Canada	Purchased organosulfur garlic compounds	DENV-2	—	Addition of *Allium sativum* compounds *abridged the level of different* pro-inflammatory cytokines including IL-8, TNF-α, IL-10 as well as iNOS (nitric oxide synthase)	Cell lines Huh-7 and U937	Hall et al. (2017)				
20	*Garcinia* × *mangostana* L./Purple mangosteen/Clusiaceae		Native to island nations of Southeast Asia and Thailand	Pericarp	DENV-1–4	α-Mangostin, 20 μM	Remarkably reduced transcription of IL-6, TNF-α (cytokine), IP-10, RANTES, and MIP-1β (chemokine)	Human hepatocellular carcinoma (HepG2), (Huh-7) and African green monkey kidney (Vero) cells	Tarasuk et al. (2017)				
21	*Schisandra chinensis* (Turcz.) Baill/Magnolia-vine, Chinese magnolia-vine, schisandra/Schisandraceae		—	Purchased compounds	DENV-1–4	Schisandrin A	Inductive efficacy of antiviral IFN-I exerts gene expression	DENV-infected Huh-7 cells	Yu et al. (2017)				
22	*Lonicera japonica* Thunb./Japanese honeysuckle/Caprifoliaceae		Native to eastern Asia	Flower buds	DENV-2	—	Subdue DENV2 multiplication *via* luciferase-reporter activity and diminishes NS1 RNA prevention level and treatment occur *via* instigation of the distinctive miRNA let-7a	Human hepatoma, baby hamster kidney (BHK-21 and *Aedes albopictus* cells C6/36)	Lee et al. (2017)				
23	*Carica papaya* L./Pawpaw/Caricaceae		Native to the tropics of the Americas but now is widely cultivated in other tropical regions	Leaves	NM	—	Level of NS1 and envelope protein decreased in the THP-1 cells, erythrocyte damage also declines	DENV-infected THP-1 cells	Sharma et al. (2019)				
24	*Schwartzia brasiliensis* (Choisy) Bedell ex Gir.-Cañas/Norantea/Marcgraviaceae		Vine native to Brazil	Leaves	DENV-2	—	Downregulated IL-6, TNF-α, IL-10, and IFN-α secretion, cellular antigenic viral load, and secreted NS1 protein reduction	Buffy coat cells and peripheral blood mononuclear leukocytes	Fialho et al. (2017)				
		Section3: Clinical studies based on animal model											
25	*Carica papaya* L./pawpaw/Caricaceae		Native to the tropics of the Americas but now is widely cultivated in other tropical regions	Leaves	—	—	Upsurge in cytokines of plasma in DENV infested AG129 mice with the dosage of freeze-dried 500 and 1,000 mg/kg	AG_129_ mice infected with DEN-2	Norahmad et al. (2019)				
26	*Cissampelos pareira* L./Abuta, ice vine/Menispermaceae		Tamilnadu, Himachal Pradesh, Bihar, west Bengal, Nagpur, Punjab, and Rajasthan	Aerial parts	DENV-1–4	Doses as high as 2 g/kg body weight for up to 1 week	—	AG_129_ mouse model	Sood et al. (2015)				
27	*Lonicera japonica* Thunb./Japanese honeysuckle/Caprifoliaceae		Native to eastern Asia	Flower buds	DENV-2	Honeysuckle (40 μl)	Up to 30% virus reduction was observed *via* suppression of DENV2 replication as well as viral titer	C57/B6 and ICR suckling mouse models	Lee et al. (2017)				
		Section 4: Clinical studies based on human model											
28	*Euphorbia hirta* L./Asthma Weed, Cats hair/Euphorbiaceae		Warmer regions of India and Australia	Plant parts not specifically mentioned–herbal water	DENV serotype not mentioned	—	Leukocyte count ominously improved from 4,000 to 11,000 mm^3^ in both female and male patients	—	Mir et al. (2012)				
29	*Lonicera japonica* Thunb./Japanese honeysuckle/Caprifoliaceae		Native to eastern Asia	Flower buds	DENV-2	—	Upregulated miRNAs–let-7a showed highest expression level	—	Lee et al. (2017)				
30	*Boerhavia diffusa* L./Tarvine/Nyctaginaceae		India, Australia, Sudan, Pakistan, Sri Lanka, South Africa, Brazil, and the southern United States	Stem	DENV serotype not mentioned	Stems of *Boerhavia diffusa* L. (10 g)	Lowers body temperature and increases platelet counts more than 85,000. Again, after 24 h, they have normal platelet count and no symptoms of dengue	—	Bharati and Sinha (2012)				
		Section 5: *In silico* molecular docking studies											
31	*Nephelium lappaceum* L./Rambutan/Sapindaceae		Southeast Asian native to the Malaysian–Indonesian region	Rind	DENV-2	—	Geraniin binds with DENV E, specifically at DIII region	—	Abdul Ahmad et al. (2017)				
32	*Glycyrrhiza glabra* L./Licorice/Fabaceae		England, Iran, Spain, Iraq, Sicily, and Russia	Not specifically mentioned –	—	—	Polyphenolic compounds, chalcones, flavonoids and some phenolics were strong docking ligands for target of dengue virus protein	—	Powers and Setzer (2016)				
33	*Psidium guajava* L./Common guava/Myrtaceae		Native to the Caribbean, Central America, and South America	Bark	DENV-2	Catechin, CC_50_ = 1,000.0; μg/ml EC_50_ = 7.8	Interactions of isolated compounds with the viral envelope protein *in silico* by docking, only naringin and hesperidin had better scores than the theoretical threshold of −7.0 kcal/mol (−8.0 kcal/mol and −8.2 kcal/mol, respectively)	—	Trujillo-Correa et al. (2019)				

## 7 Plant Extract as Mosquito Repellent and Larvicide

*Ae. aegypti* and *Ae. albopictus*, epidemiologically related arboviruses in the community health context, including Zika, dengue, and chikungunya viruses. Different types of synthetic insecticides are easily accessible in the market, associated with numerous side effects such as the development of resistance to these insecticides, having toxic effects on other organs, and ecological health problems (Rodrigues et al., 2020). However, biological control as an alternative for these vectors could be very helpful due to its eco-friendly and cost-effective nature. Govindarajan et al. (2015) reported that methanol extracts of *Delonix elata* (L.) Gamble leaves and seed (highest concentrations used: 5.0 mg/cm^2^) offered protection for over 180 and 150 min, respectively, against *Ae. aegypti.* Herbal oil formulation of different plant species including *Eucalyptus globulus* Labill., *A. indica, Mentha* × *piperita* L., *Ocimum basilicum* L., and rhizome of *Zingiber officinale* Roscoe has also been reported as repellents and bite protectors against *Ae. aegypti* and *Ae. albopictus* (Nasir et al., 2015). Terpinen-4-ol, 1,8-cineole, and *β*-pinene (0.4 mg/cm^2^) isolated from *Artemisia vulgaris* L. showed up to 91% inhibition of *Ae. aegypti* (Hwang et al., 1985). *Salvia elegans* Vahl possess acetate (11.4%), *β*-caryophyllene (6.4%), and caryophyllene oxide (13.5%), which exhibited good larvicidal activity against *Ae. aegypti* (Ali et al., 2015). Essential oils (EOs) extracted from the *Hazomalania voyronii* (Jum.) Capuron dried bark, stem, and wood are being used to attain protection against the mosquito vector *Ae. aegypti* (Benelli et al., 2020). As per WHO protocol, extracts were prepared using leaves of *Lantana camara* L.*, Hyptis suaveolens* (L.) Poit.*, Nerium oleander* L., and *Tecoma stans* (L.) Juss. ex Kunth, revealing effective larvicidal efficiency in contrast to larvae of *Ae. aegypti* (Hari and Mathew 2018). The chloroform bark extract of *Terminalia arjuna* (Roxb. ex DC.) Wight and Arn. showed maximum mortality on *Ae. aegypti* larvae. The LC_50_ and LC_90_ values of *T. arjuna* on *Ae. aegypti* larvae were 4.61 and 24.17 µg/ml, respectively (Tamilventhan and Jayaprakash 2019).

## 8 Some Phytoconstituents With Anti-Dengue Activity and Their Action Mechanism

Numerous plants and plant-based products exhibit enormous biological properties (like an antibiotic, antitumor, and antiviral), which are responsible for treating millions of individuals with serious diseases. Among those flavonoids, polysaccharides, hemicelluloses, and hydrophilic colloids are involved in the antiviral properties of plants (Table 3). Some of these biologically active compounds are based on recent research, and their promising role as contemporary medication in the near future has been discussed briefly.

**TABLE 3:** Effective bioactive compounds from plant sources against dengue infection.

Sr. no	Phytochemical	Chemical structure	Class	Plant studied for anti-dengue activity	Plant Part used	Other plants containing this phytoconstituent	Citation
1	Castanospermine	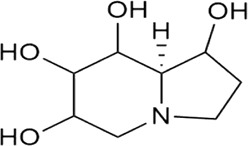	Tetrahydroxyindolizidine alkaloid	*Castanospermum australe* A. Cunn. and C. Fraser (Fabaceae)	—	1. *Swainsona canescens* (Lindl.) F. Muell. (Fabaceae)	Häusler et al. (2000); Whitby et al. (2005)
2	Baicalin	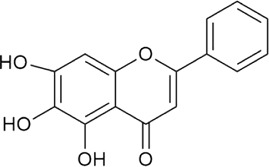	Glycosyloxyflavone	*Scutellaria baicalensis* Georgi (Lamiaceae)	Roots	1. *Scutellaria baicalensis* Georgi (Lamiaceae) inhibit human T-cell leukemia virus type 1(HTLV-I)	Baylor et al. (1992); Zhao et al. (2016); Zhou et al. (2016); Rojsanga et al. (2020)
						2. *Oroxylum indicum* (L.) Kurz (Bignoniaceae)	
3	Gallic acid	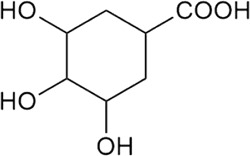	Phenolic acid	*Psidium guajava* L. (Myrtaceae)	Bark	1*. Camellia sinensis* (L.) Kuntze (Theaceae)	Trujillo-Correa et al. (2019); Zhou et al. (2020); Baite et al. (2021)
						2*. Ficus auriculata* Lour. (Moraceae)	
4	Quercetin	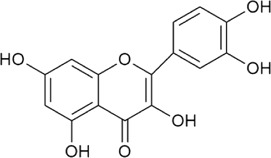	Flavonoid glycosides	*Momordica charantia* L. (Cucurbitaceae)	—	1*. Allium roseum* L. (Amaryllidaceae)	Han et al. (2005); Eid et al. (2010); Agrawal and Pal (2013); Nile et al. (2017)
						2*. Vaccinium vitis-idaea* L*.* (Ericaceae)	
						3. *Nyctanthes arbor-tristis* L. (Oleaceae)	
5	Epigallocatechin gallate	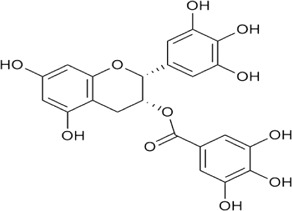	Polyphenols	*Camellia sinensis* (L.) Kuntze (Theaceae)	—	—	Vázquez-Calvo et al. (2017)
6	Galactomannan	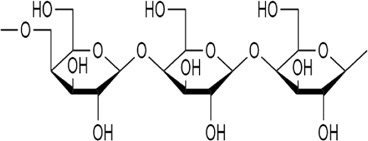	Polysaccharides	*Leucaena leucocephala* (Lam.) de. Wit (Fabaceae), *Mimosa scabrella* Benth. (Fabaceae)*, Lippia alba* (Mill.) N.E.Br. ex Britton and P. Wilson (Verbenaceae)	Seed	1. *Trigonella foenum-graecum* L*.* (Fabaceae)	Latgé et al. (1991); Chaubey and Kapoor (2001); Ono et al. (2003); Mathur and Mathur (2005); Ocazionez et al. (2010); Prajapati et al. (2013)
						2. *Ceratonia siliqua* L. (Fabaceae)	
						3. *Cyamopsis tetragonoloba* (L.) Taub. (Fabaceae)	
						4. *Caesalpinia spinosa* (Molina) Kuntze (Fabaceae)	
						5. *Senna alexandrina* Mill. (Fabaceae)	
7	Glabranine, 7-O-methylglabranine	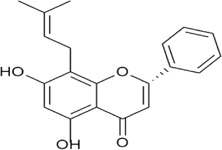	Flavonoid	*Tephrosia* species	Leaf, Flower	1. *Linum usitatissimum* L. (Linaceae)	Sánchez et al. (2000)
8	Galactan	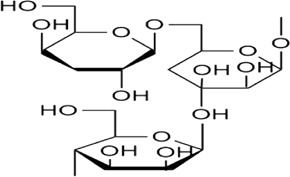	Hemicelluloses	*Cryptonemia crenulata* J. Agardh (Halymeniaceae)*, Gymnogongrus torulosus* (J.D. Hooker and Harvey) F. Schmitz (Phyllophoraceae)	Whole Plant	1.*Chenopodium quinoa* Willd (Amaranthaceae)	Talarico et al. (2005); Wefers et al. (2014)
9	Kappa carrageenan	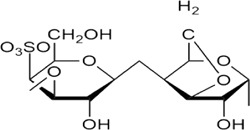	Hydrophilic colloids	*Meristiella gelidium* (J. Agardh) (Solieriaceae)*, Gymnogongrus griffithsiae* (Turner) C. Martius (Phyllophoraceae)	—	1*. Hypnea musciformis* (Wulfen) J.V. Lamouroux (Cystocloniaceae)	Talarico et al. (2005); Arman and Qader (2012); Nur Fatin Nazurah and Nur Hanani (2017)
						2. *Eucheuma cottoni* Weber-van Bosse (Solieriaceae)	
10	4-Hydroxypanduratin A, panduratin A	—	—	*Boesenbergia rotunda* (L.) Mansf. (Zingiberaceae)	—	1. *Boesenbergia rotunda* (L.) *Mansf.* (Zingiberaceae)	Kiat et al. (2006); Pham et al. (2020)
11	Zosteric acid	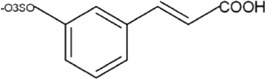	Flavonoid	*Zostera marina* L. (Zosteraceae)	—	—	Rees et al. (2008)
12	Morin	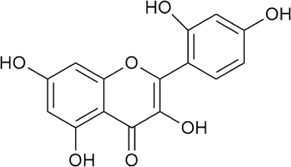	Flavonoid	*Zingiber officinale* Roscoe (Zingiberaceae)	—	1. *Tinospora crispa* (L.) Hook. f. and Thomson (Menispermaceae)	Yang and Lee (2012); Hussain et al. (2014); Mbadiko et al. (2020); Taguchi et al. (2020); Warsinah et al. (2020)
						2. *Morus alba* (L.) (Moraceae)	
						3. *Acridocarpus orientalis* A. Juss. (Malpighiaceae)	
13	Hyperoside	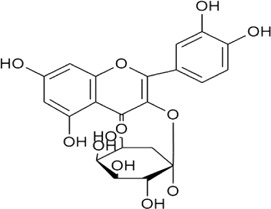	Flavonoid	*Houttuynia cordata* Thunb. (Saururaceae)	Whole plants, aerial stem and leaves	1. *Camptotheca acuminata* Decne. (Cornaceae)	Li et al. (2005); Kim et al. (2011); Leardkamolkarn et al. (2012); Ahn and Lee (2017); Zhang et al. (2021)
						2. *Hypericum perforatum* L. (Hypericaceae)	
						3. *Oenanthe javanica* (Blume) DC. (Apiaceae)	
						4. *Zanthoxylum bungeanum* Maxim. (Rutaceae)	
14	Fucoidan	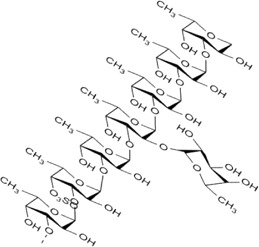	Polysaccharides	*Cladosiphon okamuranus* Tokida (Chordariaceae)	Whole plants	1*.Utricularia aurea* Lour*.* (Lentibulariaceae)	Jiang et al. (2010); Skriptsova et al. (2010); Marudhupandi and Kumar (2013); Minh Ly et al. (2017); Palanisamy et al. (2017); Lim et al. (2019)
						2*. Undaria pinnatifida* (Alariaceae)	
						3. *Sargassum wightii* Greville ex J. Agardh (Sargassaceae)	
						4. *Sargassum polycystum* C. Agardh (Sargassaceae)	
						5. *Ascophyllum nodosum*	
						(L.) Le Jolis (Fucaceae)	
						6. Vietnam Sargassum species	
15	Daidzein	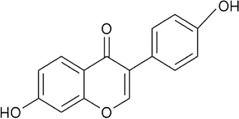	Bioflavonoid	—	—	1*. Glycine* max (L.) Merr. (antioxidant) (Fabaceae)	Pongkitwitoon et al. (2011); Juliana et al. (2020)
						2. *Pluchea lanceolata* (DC.) C.B. Clarke (Asteraceae)	
						3*. Pueraria candollei* Benth*.* (Fabaceae)	

### 8.1 Castanospermine

Castanospermine (a natural alkaloid) is a derivative of *Castanospermum australae* A. Cunn. and C. Fraser (black bean) (Whitby et al., 2005) that can easily be isolated through simple purification methods (Pan et al., 1993). One of the possible mechanisms through which it prevents infection is misfolding of viral proteins *via* obstructing the removal of glucose residues from N-linked glycans (Whitby et al., 2005). This may further lead to inhibit molecular interaction of viral and host proteins (Zhang et al., 1997; Molinari and Helenius 1999). Castanospermine has shown not only inhibitory effects on all the dengue serotypes by preventing the production of DENV but also evidence to prevent mice death when infected with DENV-2 through the intracranial route (Rathore et al., 2011). Evidence of celgosivir (6-O-butanoyl castanospermine) being an antiviral agent against DENV virus both *in vitro* and *in vivo* has also been concluded. Castanospermine exhibits glucosidase inhibitor properties that result in early-stage inhibition of glycoprotein processing. As a result, the formation of unstable complexes produces non-productive viral protein (prM and E) folding pathways and ultimately the antiviral response of Castanospermine (Courageot et al., 2000). Celgosivir is the butylated prodrug cleaved in cells to produce castanospermine, a bicyclic iminosugar (Miller et al., 2018). It causes misfolding and accumulation of NS1 (DENV non-structural protein) in the endoplasmic reticulum and also alters host protein response leading to the antiviral activity. Based on the previous research and data availability, castanospermine use in anti-dengue medication can be an important contribution in fighting dengue infection (Rathore et al., 2011).

### 8.2 Baicalin

Baicalin (a flavonoid) demonstrates considerable antiviral potential against DENV-2 by targeting the replication stages post-viral infection (Zandi et al., 2012). Baicalein is reported to be isolated naturally from the roots of *Scutellaria baicalensis* Georgi, a medicinal plant of China, and can be further converted into baicalin after intake by animals or humans (Xu et al., 2010). Based on the successful results obtained from Baicalin against dengue infection, other plant species having baicalin as a biomolecule can be explored for their anti-dengue properties. The mechanistic approach behind the anti-dengue properties has been demonstrated by researchers where possible ways of antiviral activity (96–99%) are direct inactivation of free DENV-2 particles, inhibited intracellular viral replication, and binding of DENV-2 cells to the host cell (Moghaddam et al., 2014). In molecular docking analysis, both baicalin and baicalein are reported to inhibit the DENV replication by pursuing key DENV genes and exhibit anti-DENV protease (NS2B/NS3) activity. The results of docking studies show that both baicalein and baicalin may interact with NS3–NS2B, E, and NS5 proteins, which can be responsible for their antiviral impact (Hassandarvish et al., 2016; Rasool et al., 2019). Further investigation using these phytoconstituents is still needed to obtain promising results in the form of contemporary medications and future implementation in clinical use.

### 8.3 Gallic Acid

Gallic acid (main phenolic plant component) exhibits various pharmacological activities (antioxidant, antiviral, antifungal, etc.) and therapeutic uses against neural disorders, dengue, cancer, asthma, and allergic rhinitis, without any cytotoxic effects (Suganthi and Ravi 2019). In a study, inhibitory response of gallic acid with no cytotoxic effect has been elucidated in *in vitro* conditions on DENV-2 virus along with another compound, emodin, and some plant extracts, i.e., *Antennaria microphylla* Rydb.*, Rubus scaber* Weihe, *Ziziphus jujuba* Mill., and *Commelina benghalensis* L. Gallic acid was found to be active against dengue disease through prophylactic treatment rather than post-infection medication (Batool et al., 2018), while another report has suggested successful viral inhibition in both prophylactic (52.6%) and post-treatment (67.3%) approaches (Trujillo-Correa et al., 2019). More complete investigations are warranted on these plants for isolation, purification, and characterization of bioactive principles responsible for the anti-dengue activity, and to elucidate their underlying mechanisms of DENV inhibition (Batool et al., 2018). Moreover, isobutyl gallate (a derivative of gallic acid) due to low cellular toxicity and antiviral response at low concentration has been suggested as a good alternative for anti-DENV activity (by inhibiting viral replication) (Dewi et al., 2019). Considering all mentioned findings and inconsistencies about cellular toxicity and anti-dengue effects, more clarification is still needed, which can lead to formulating a successful treatment against the DENV.

### 8.4 Quercetin

Quercetin (flavonoid) exhibits various pharmacological responses against viruses, tumors, inflammation, etc. In accordance with previous studies available, quercetin is one of the main components of different plant extracts (*E. hirta* and *P. guajava*) exerting consistent antiviral effect against dengue infection (DENV-2) (Zandi et al., 2011; Saptawati et al., 2017; Suganthi and Ravi 2019). A possible mechanism is similar to the previously discussed metabolites that inhibit viral replication by targeting RNA polymerase enzyme. Quercetin is reported to exhibit antiviral properties *via* impeding viral attachment and viral replication against DENV serotypes (1–4) (Zandi et al., 2011; Sharma et al., 2018). In addition, quercetin is one of the main components present in *P. guajava* leaf extract demonstrating the inhibitory effect on DENV infection with no involvement in the inhibition of viral surface proteins and receptors. These findings also suggest the presence of other bioactive compounds in *P. guajava* leaf formulations that may exhibit anti-dengue behavior (Dewi et al., 2020). *Houttuynia cordata* Thunb. leaves ethyl acetate fraction (including quercetin, quercitrin, and rutin) has also been reported, which reveals the efficient anti-DENV-2 activity of bioactive components quercetin (IC_50_ of 176.76 μg/ml) and quercitrin (IC_50_ of 467.27 μg/ml). The synergistic effect of both components is also depicted in the past presenting antiviral properties with an IC_50_ of 176.76 μg/ml (Chiow et al., 2016). These results conclude that plant formulations and their metabolites signify a promising preventative substance in *P. guajava* L. leaf extract, which might prevent DENV attachment by inhibition of DENV surface protein along with the receptor inhibition (Dewi et al., 2020).

### 8.5 Epigallocatechin Gallate

Epigallocatechin gallate (flavonoid) and delphinidin (structurally related polyphenol) from plants and their products (wine, green tea, curcumin, and delphinidin) have proved their antiviral qualities regardless of the DENV serotypes. These compounds are suggested to exhibit virucidal effects or inhibit viral production by acting upon the viral life cycle at its attachment and entry points (Vázquez-Calvo et al., 2017; Raekiansyah et al., 2018; Clain et al., 2019). In another study, epigallocatechin gallate, a biologically active component of green tea, curcumin, and delphinidin, is reported to have the least cytotoxic effects. It is believed to prevent viral infection at early stages by direct interaction with the virion (when the virus is preincubated with epigallocatechin gallate), which leads to an approximate drop of 90% in DENV antigen present in intracellular fluid. Similarly, epigallocatechin, another bioactive compound, also has a strong repressive impact on viral growth (Raekiansyah et al., 2018). The inhibition mechanism as suggested by Raekiansyah et al. is to develop a virucidal effect through direct binding with molecules present on the DENV surface (Raekiansyah et al., 2018).

### 8.6 Other Bioactive Compounds

Apiofuranoside (flavanone glycosides), obtained from *Faramea bahiensis* Mull. Arg., is efficiently involved in the treatment of dengue *via* controlling viral replication as well as reducing the number of infected cells (12%) and RNA copies of DENV-2 (67%) in HepG2 cells (Nascimento et al., 2017). Flacourtoside A, a phenolic glycoside exhibited in *Flacourtia indica* (Burm.f.) Merr., effectively inhibited DENV replication with the 9.3 µM IC_50_ values (Bourjot et al., 2012). Trigocherrin A and Trigocherriolide B and C (diterpenoids) found in *Trigonostemon cherrieri* Veillon are also able to impede DENV replication with 12.7 µM and 3.1 and 16.0 µM IC_50_ values, respectively (Allard et al., 2012). *Arrabidaea pulchra* (Cham.) L.G. Lohmann synthesizes another important bioactive compound, verbascoside (phenyl glycoside), which is reported to display good anti-DENV-2 (IC_50_ = 3.4 μg/ml) properties (Brandão et al., 2013). Also, pectolinarin, a type of flavone found in *Amphilophium elongatum* (Vahl) L.G. Lohmann exhibited anti-DENV-2 effects with an EC_50_ value of 86.4 μg/ml (Simões et al., 2011).

Note that the action mechanism and the minimal cytotoxicity levels of natural compounds can contribute to the development of anti-dengue medication, which will be safer as well as more effective in use, although limited research has been done in this aspect and there are limited publications. Thus, further investigations are necessary to develop a fruitful antiviral drug (to cure DENV).

## 9 Immunomodulatory Response of Plant Extracts

With the onset of dengue infection, the host can experience a complex interplay of host immune factors and viral particles, which may include amplified immune cell infection due to the presence of non-deactivating antibodies and stimulation of cross-reactive autoimmunity responses (i.e., activation of T cells and autoantibodies, cytokine deregulation, complement, and coagulation systems) (Jasso-Miranda et al., 2019). Being an acute febrile infection (thrombycytopenia, arthralgia, and hemorrhagic symptoms), it may lead to the host deterioration due to the hemorrhagic attack, plasma leak, intense shock, organ collapse, and ultimately, death (Pan American Health Organization (PAHO) 2016; Jasso-Miranda et al., 2019). Dengue infection is hypothesized to be more fatal due to the antibody-dependent enhancement of infection, which is associated with the transformation of the disease. Two DENV infections in a specific sequence, the interval of two infections, and how much the human host is causative in terms of age, health, ethnicity, and genetic background (Guzman et al., 2013). Generation of cross-reactive antibodies, post-first-time dengue infection in association with the second infecting virus, results in the formation of more harmful immune compounds like non-deactivating antibodies (critical players of the pathogenic response in severe dengue conditions) (Kuczera et al., 2018). This consequently increases the infected cell count and thus increased viral output in the form of high cytokine production, which further causes vascular permeability, intense shock, and, ultimately, death (Puerta-Guardo et al., 2013). Various other immune factors like interferons (IFN-α and -γ), interleukin (IL-6, -8, and -10), and tumor necrosis factors (TNF-α), and elevated expression of cytokine signaling suppressors are linked to severe dengue infections (Bozza et al., 2008; Chen et al., 2014; Flores-Mendoza et al., 2017). Another study revealed a potential immunomodulatory response (against DENV virus) by *Schwartzia brasiliensis* (Choisy) Bedell ex Gir.-Cañas Choisy extract, which helps in antigen load reduction (NS1 protein, a viral load indicator) in the cells. Crude *S. brasiliensis* extract was most efficient against dengue activity while the dichloromethane fraction resulted in strong immunoregulatory activities. Downregulation of various immune factors like TNF-α, IFN-α, IL-6, and IL-10 was evidenced as the key reason behind the antiviral effects (Fialho et al., 2017). Significant immunoregulation and antiviral response with *Uncaria tomentosa* (Willd ex Schult.) DC. fractions have also been demonstrated, in which DENV-infected monocytes (human) were tested. The anti-DENV activity was associated with decreased cytokine levels such as TNF-α and modulation of IL-10 (Reis et al., 2008). Successful molecular assessment and manipulations of various immune responses and associated factors can be useful research to develop anti-dengue drugs. Traditionally, *C. papaya* leaf extract has been proven for its immunomodulatory activities (Pandey et al., 2016; Anjum et al., 2017; Razak et al., 2021). In dengue patients (suffering from thrombocytopenia), oral administration of papaya leaf extract has resulted in increased platelet count activity (40–48 h) (Subenthiran et al., 2013). In other similar findings with the use of *C. papaya* leaf extract, beneficial impacts are reported where a significant increase in WBC and platelet count is evidenced with negligible side effects (Hettige 2008; Gowda et al., 2015). *Rhodiola imbricata* Edgew. (a flowering plant) is also demonstrated to induce pharmacological modifications in response to the DENV infection. It induces NK cells and cytokines like interferon b (IFN), IL-8, IL-1b, IL-6, and TNF-a and upregulates phosphorylated NF-kB, eIF-2a, and PKR in DENV-infected cells (Diwaker et al., 2014). In addition, the immunomodulatory potential against DENV is stated by its ability to regulate cytokine production, phagocytic activity, and white blood cell proliferation (Razak et al., 2021).

## 10 Complementary and Alternative Dengue Therapeutics

Dengue vaccines have been classified into five main categories, i.e., live attenuated vaccines (CYD-TDV, TV003/TV005), DENVaxin activated virus vaccine (PIV), recombinant subunit vaccine (V180), DNA vaccine (D1ME100, TVDV), and viral vectored vaccine (TLAV Prime/PIV boost and reverse order) (Deng et al., 2020a). Dengvaxia (CYD-TDV) by Sanofi Pasteur (the first dengue vaccine) was registered in several endemic countries for individuals of the age group 9–45 years (Deep et al., 2018; Deng et al., 2020a). It consists of DENV (1–4) serotype structural genes (encoding PrM and E proteins) introduced in the attenuated yellow fever vaccine strain genome. According to the reports, it exerts 56%–61% virucidal efficacy against dengue in Asia and Latin America (Capeding et al., 2014; Biswal et al., 2019). This anti-dengue vaccine is only recommended for patients having evidence of past DENV infection due to infection severity in seronegative candidates *via* stimulating non-neutralizing antibody generation (Sridhar et al., 2018). This drawback is considerable in the development of other alternatives of anti-dengue drug discovery. Another tetravalent dengue medicine, TAK-003 (Takeda), consisting of live attenuated DENV-2 genetic backbone for all DENV serotypes, has been designed by experts at the Division of Vector-Borne Diseases, Centers for Disease Control and Prevention (CDC) (Huang et al., 2013). Overall vaccine efficiency is documented to be 80.9%, and the seronegative populations showed 74.9% vaccine efficacy for the new dengue vaccine (TAK-003) (Biswal et al., 2019). The vaccination priming with drugs used in combination has been reported to provide more effective immunogenic protection in animals. In addition, analysis of the immune response and the mechanism of viral elements can be a revolutionary success in vaccine development (Liu et al., 2016). Anti-dengue drugs, such as chloroquine, celgosivir, balapiravir, prednisolone, and lovastatin, have been stated to undergone medical examinations against DENV infection (Kaptein and Neyts 2016; Low et al., 2017), although no effective dengue treatment has been developed from any of these composites. Therefore, the search for more effective antiviral compounds (plant-derived or second-use medicines) is still a vital prerequisite in overcoming dengue’s lethal effects (Trujillo-Correa et al., 2019). Besides, the specific anti-dengue vaccine is not yet developed, as few to none of the potential anti-dengue candidates have been tested clinically (Razak et al., 2021).

## 11 Conclusion

An update about various plants with pharmacological and ethnobotanical uses against dengue treatment was presented. Besides the global status of the dengue epidemic, existing vaccination measures have been conferred in the current review. Solely, the folkloric knowledge and uses of natural resources have been promising due to the exhaustive investigation carried out on the ethnopharmacologically important plant species. Many plant species and their respective extracts and pure compounds have been shown to exhibit potential as anti-DENV medicaments. The plant bioactive metabolites exhibit antiviral response directly or through the stimulation of immunomodulatory response cascades against DENV at different infection stages, i.e., viral adsorption, intracellular replication, and proliferation. Plant metabolites also help to reduce the antigen load and manipulate various immune factors in the host. Plant-mediated immunomodulation leads to the regulated cytokine production, enhanced platelet count, and phagocytic pathway activation. However, there are several plants still used for their anti-dengue properties *via* traditional methods and are yet to be investigated for scientific approval. Whether the antiviral properties are a result of a single phytoconstituent or the interaction of multiple phytoconstituents present in the plant extract(s) is critical to justify. Thus, extensive research is required to assess the immunologic potentials along with the phytochemical richness of plants and further clinical probes to develop an effective medicine. Another significant aspect of medicinal plants is their insecticidal properties, which make them an eco-friendly and sustainable substitute for *Aedes* mosquito control. Since vaccine development is a time-consuming process, finding an alternate treatment is crucial to overcome the lethal impacts of the DENV virus. Plant natural compounds are valuable sources to accomplish the speedy discovery of anti-DENV drugs because of their safer use and positive immunomodulatory responses. Nevertheless, the most crucial task is to reveal the molecular machinery of viral components and their capability to mutate rapidly during replication. Moreover, there are some vaccines available to treat dengue infection, but with some limitations. In this scenario, research is being carried out on some medicinal plant species, i.e., *Nyctanthes arbor-tristis* L., *Firmiana simplex* (L.) W. Wight, *T. sinensis*, and *Moringa oleifera* Lam., to investigate their metabolic profiles supporting their anti-dengue properties (antilarval or anti-DENV, in the authors’ laboratory). Besides, *N. arbor-tristis*, *M. oleifera*, and *T. sinensis* are also reported for their larvicidal activities. Furthermore, the GC-MS profiling of *F. simplex* also reveals the presence of natural compounds having insecticidal properties against dengue vectors.

Besides, a lot of scientific research is dedicated towards anti-DENV drug development and further research is needed to identify phytochemicals for their antiviral efficacy, mode of action, and successful clinical implementation.
